# Effects of exergaming on executive function of older adults: a systematic review and meta-analysis

**DOI:** 10.7717/peerj.13194

**Published:** 2022-04-11

**Authors:** Jiahui Jiang, Wei Guo, Biye Wang

**Affiliations:** 1 College of Physical Education, Yangzhou University, Yangzhou, China; 2 Institute of Sports, Exercise and Brain, Yangzhou University, Yangzhou, China

**Keywords:** Exergaming, Executive function, Older adults, Meta-analysis

## Abstract

**Background:**

Executive function (EF) involves a series of high-level processes, such as inhibition, switching, and updating. Aging-related cognitive decline has been shown to be strongly associated with EF worsening. The aims of this study were to perform a meta-analysis to evaluate the effects of exergaming, an emerging intervention, on EF performance in older adults and to conduct a moderator analysis of exergaming effects on EF.

**Methods:**

Randomized controlled trials examining exergaming influences on EF in older adults were collated by searching the Web of Science, Elsevier Science, PubMed, and Google Scholar databases. Statistical data were quantified in Comprehensive Meta-analysis software. Overall EF and EF domains (inhibition, switching, and updating) were analyzed separately.

**Results:**

A total of 15 studies were included. The meta-analysis results indicated that exergaming had a significant influence on overall EF in the older adult (standardized mean difference (SMD) = 0.349, 95% confidence interval (CI) [0.191–0.506], *p* < 0.001). The same effects were also found in EF domains of inhibition (SMD = 0.415, 95% CI [0.102–0.729], *p* = 0.009), switching (SMD = 0.243, 95% CI [0.071–0.415], *p* = 0.005), and updating (SMD = 0.366, 95% CI [0.140–0.592], *p* = 0.002). The effects of exergaming on overall EF were found to be moderated by the frequency of the intervention (Q_(1)_ = 3.537, *p* = 0.06).

**Conclusion:**

Exergaming was confirmed to improve overall EF, as well as in older adults, and the effect of exergaming on EF was shown to be moderated by intervention frequency.

## Introduction

By 2050, the global population of people aged 65 and older is expected to more than double to 1.5 billion people, at which time approximately one in six people in the world will be over 65 (United Nations Department of Economic & Social Affairs, Population Division, 2020). Cognitive aging has been shown to be an important cause of work and life limitations (Fillit et al., 2002). Executive function (EF) involves a series of high-level processes, such as inhibition, switching, and updating (Collette et al., 2006). Declining EF in older adults has been shown to be a main cause of cognitive aging and to be disruptive to daily living (Braver & West, 2015). Thus, maintaining or improving EF in older people may be an effective means of countering cognitive aging.

Non-pharmacological interventions, especially forms of physical exercise, *e.g*., aerobic exercise (Colcombe et al., 2006; Frith & Loprinzi, 2018) and resistance training (Liu-Ambrose et al., 2010; Anderson-Hanley, Nimon & Westen, 2010), have been associated with positive effects on cognitive performance in elderly people. Video game play has also emerged as an interesting cognitive intervention for improving cognitive function in older people (Basak et al., 2008; Peretz et al., 2011; Anguera et al., 2013). Moreover, combining physical and video interventions may be more effective than either single intervention alone (Bamidis et al., 2015). Exergaming, defined as any type of video game play that requires players to engage in whole body movements (Gerling & Mandryk, 2014), is an emerging simultaneous intervention that combines physical exercise with a video game. Previous meta-analyses suggested that simultaneous training benefits cognition more than sequential training (Zhu et al., 2016; Gheysen et al., 2018). Such findings suggest that exergaming represents a promising method for improving the course of cognitive aging.

Although there has been substantial researches examining the influence of physical exercises (Leyland et al., 2019; Chen et al., 2020) and video games (Durlach, Kring & Bowens, 2009; Cain, Landau & Shimamura, 2012) on EF performance, there are limited data regarding the effects of exergaming on EF, especially in older adults, and the available data are controversial (Barcelos et al., 2015; Ordnung et al., 2017). Prior studies that have examined the effects of exergaming in older adults have focused primarily on balance (Whyatt et al., 2015; Padala et al., 2017), postural control (Merriman et al., 2015; Sato et al., 2015), and quality of life (Collado-Mateo et al., 2017; Schumacher et al., 2018). Relatively few studies have focused on exergaming effects on EF (Barcelos et al., 2015; Anderson-Hanley et al., 2018) and fewer still on specific domains of EF (Monteiro-Junior et al., 2017b; Phirom, Kamnardsiri & Sungkarat, 2020), as opposed to EF as a whole.

Two meta-analyses have examined the effects of exergaming in older adults. One explored the effects of exergaming on balance, and showed significant effect on dynamic balance, perceived balance, Chair Stand Test, and balance test batteries (Fang et al., 2020). While the other founded that exergaming is less effective on postural control when measured using rating scales, distance-based reaching tasks, balance confidence and fear of falling, as compared to alternative balance training modes (Tahmosybayat et al., 2017). To our knowledge, there have not been any that have examined the effects of exergaming on EF in older adults. Furthermore, among the prior studies that have been reported on the effects of exergaming on EF in older adults, there are inconsistencies among them in terms of intervention design (*e.g*., intervention duration, session frequency, session duration) (Schättin et al., 2016; Adcock et al., 2020; Phirom, Kamnardsiri & Sungkarat, 2020), cognitive status of the subjects (healthy or mild cognitive impairment (MCI)) (Guimarães, Barbosa & Meneghini, 2018; Liao et al., 2019) control groups (active or passive) (Schättin et al., 2016; Adcock et al., 2020). These factors have been shown to potentially influence intervention effects (Zhu et al., 2016; Guo et al., 2020; Ruiz-González et al., 2021). Furthermore, type of exergaming was also considered as a potential factor that could influence the result of intervention. Therefore, the present study classified exergaming into customized and off-the-shelf types (Gschwind et al., 2015). Customized exergaming refers to the system specifically tailored based on research purposes, such as iStoppFalls and Active @ Home system (Gschwind et al., 2015; Adcock et al., 2020). Off-the shelf exergaming, on the other hand, is not sufficiently tailored to the specific circumstances and values, such as Nintendo Wii and Microsoft Xbox system (Maillot, Perrot & Hartley, 2012; Guimarães, Barbosa & Meneghini, 2018). In additional, the two meta-analyses mentioned above didn’t explore the impact of these factors on the results (Tahmosybayat et al., 2017; Fang et al., 2020). Therefore, the aims of the current study were to perform a meta-analysis evaluating the effects of exergaming on EF performance in older adults and to conduct a moderator analysis of participant and intervention characteristic variables.

## Materials and Methods

This review was registered with the International Prospective Register of Systematic Reviews (PROSPERO, CRD42021290137) and reported in accordance with the Preferred Reporting Items for Systematic Reviews and Meta-Analysis (PRISMA; Moher et al., 2010).

### Search strategy

Systematic literature searches were conducted in the PubMed, Web of Science, Elsevier Science and Google Scholar databases through February 2021. The following keywords were used: intervention terms (“exergaming” OR “exergame” OR “active video game” OR “active video gaming” OR “virtual reality exercise” OR “virtual video gaming” OR “virtual video game” OR “interactive physical and cognitive” OR “interactive physical-activity video-game”), AND cognitive terms (“cognitive function” OR “cognition” OR “executive function” OR “inhibition” OR “switching” OR “updating”), AND subjects population terms (“older adult” OR “elderly” OR “aging” OR “mild cognitive impairment”). Additionally, the reference lists of the retrieved articles were reviewed manually to identify additional relevant articles. Two independent reviewers (J. Jiang and W. Guo) did the screening based on titles and abstracts initially. The remaining articles were further screened by full-text assessment. Any disagreements between the two reviewers were resolved through consensus and by discussion with a third author (B. Wang).

### Eligibility criteria

Studies were included for this meta-analysis if the following eligibility criteria were met: (1) randomized controlled trial design; (2) sample population of older adults who could perform daily activities with no or minimal assistance and who had normal vision and no or minimal neurological deficits or cognitive impairments; (3) exergaming platform intervention employed; (4) none exergaming intervention for control group (5) enough information reported to calculate an effect size for at least one EF outcome measure; and (6) written in English. The following types of studies were excluded: (1) non-intervention studies; (2) non-randomized studies; (3) editorial or conference abstracts; (4) book chapters; (5) review articles or theoretical articles; and (6) unpublished studies, abstracts, or papers.

### Data extraction and analysis

EF domains were classified based on a preview study (Collette et al., 2006). Inhibition ability was represented with performance in Flanker, Stroop, Go/no-go task, Executive control, Response inhibition tests. Switching ability was represented with performance in the Color trails, Set-shifting and Trail making tests. Updating ability was represented by performance in Digit Span, Floor maze, Digit-symbol substitution and N-back tests. All of the above tasks were included in an assessment of overall EF.

The EF outcome data are derived by extracting (1) means, standard deviations (SDs) and the number of participants in each group at pre- and post-intervention or (2) mean change and SDs difference and the number of participants in each group. All data were then synthesized quantitatively in Comprehensive Meta-analysis software. An accurate test for homogeneity of variation in effect sizes across studies based on Q-statistic. The proportion of true heterogeneity in observed variance was assessed by the I^2^-statistic. The I^2^-statistic was used to evaluate the heterogeneity of the included studies. If heterogeneity test showed *p* ≥ 0.05 and I^2^ < 50%, representing no statistical heterogeneity among the studies, then fixed-effects model would be used for analysis. If *p* < 0.05 and I^2^ ≥ 50%, a random-effects model would be used for analysis. The Egger’s regression intercept test was used to estimate publication bias (Stuck, Rubenstein & Wieland, 1998). Moderating variable analysis was used in this study to explore the potential factors which would affect the effect. In this study, the intervention effect was measured in terms of standardized mean difference (SMD) between the experimental and control group (Borrenstein et al., 2009). Positive SMDs indicates intervention effectiveness. The pooled SMD was computed by averaging the effect sizes of all tasks in each study. When SMD of each study was obtained, a combined effect size with 95% confidence interval (CI) was calculated to determine the efficacy of exergaming. The effects of exergaming on EF in older adults was evaluated by the combined effect size and 95% CI of all corresponding studies.

### Evaluation of methodological quality

The methodological quality of the included articles was evaluated independently by two reviewers. Physiotherapy Evidence Database (PEDro) scale was used to rate the quality of the eligible studies (Maher et al., 2003). Disagreements on ratings were resolved through consensus or by discussion with B. Wang before a final decision was made. The range of potential quality scores for individual articles was 0–11 with the following cut-off definitions: ≥10 points, high methodological quality; 7–9 points, medium methodological quality; and <7 points, low methodological quality (Verhagen et al., 2007; Viswanathan et al., 2017).

## Results

**Figure 1:**
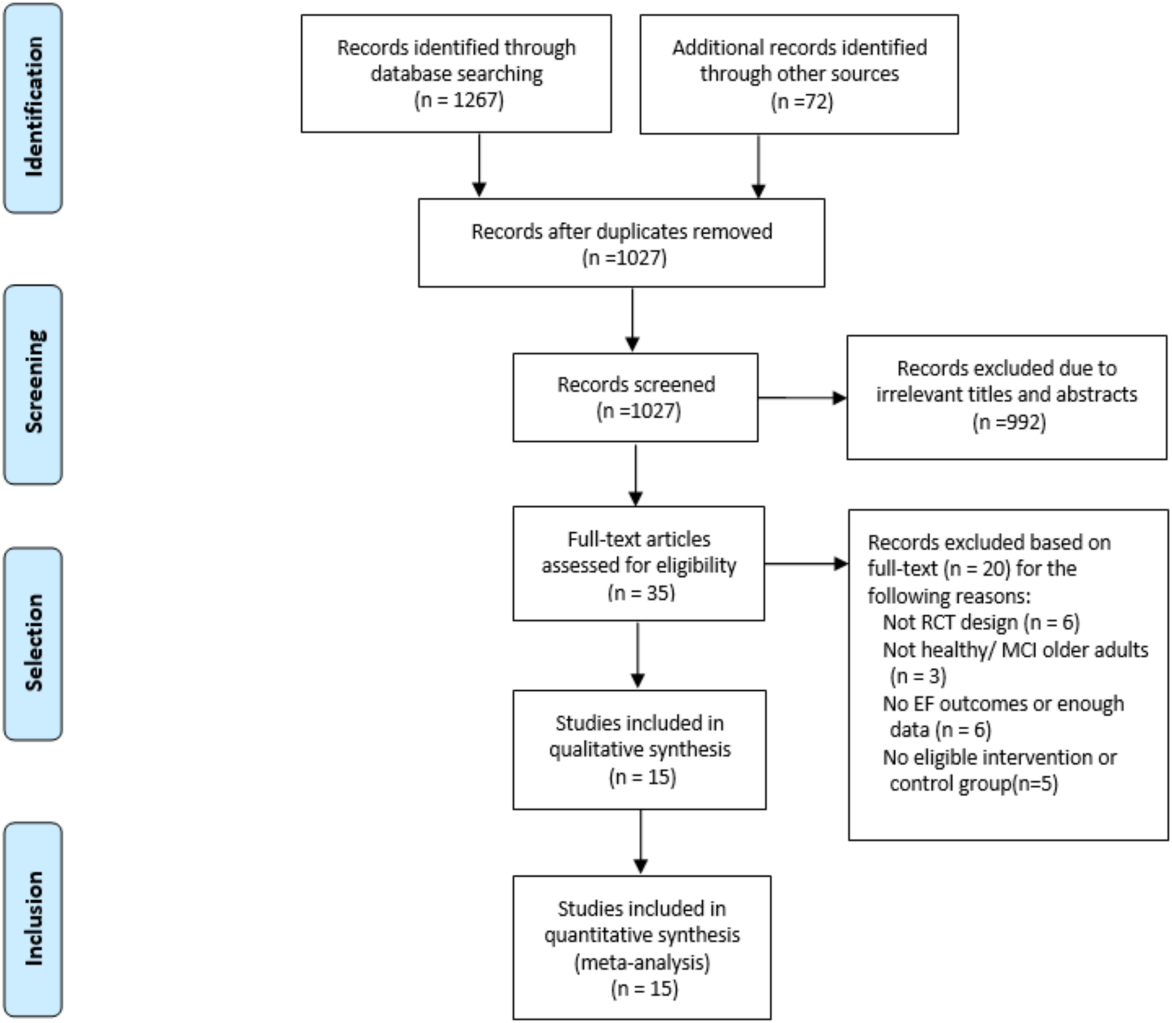
Selection process for the meta-analysis.

Ultimately, 15 eligible studies, with a combined total of 650 participants, were included in this meta-analysis. An overview of the specific selection process is provided in Fig. 1. Among these studies, 11 enrolled healthy older adults and 2 enrolled older adults with MCI. The mean age of participants ranged from 60.4 years to 86.0 years. Eleven of the included studies used an active control group design, and four used a passive control group design. The main characteristics of the 15 articles are summarized in Table 1.

**Table 1:** Main characteristics of the included studies.

Study	Sample size	Population	Mean age	Session content	Frequency	Comparison	EF measure tasks	Study quality
Anderson-Hanley et al. (2012)	63	H/MCI	78.7	Cybercycle	12 weeks, 3 × 45 min per week	Traditional stationary bike	Color Trails	7
Maillot, Perrot & Hartley (2012)	32	H	73.5	Nintendo Wii	24 weeks, 2 × 60 min per week	Sedentary lifestyle and not playing video exergaming	Stroop Trail Making Digit-Symbol	6
Eggenberger et al. (2016)	33	H	74.9	Interactive video game dancing	8 weeks, 3 × 30 min per week	Balance and stretching training	Trail Making Executive Control Stroop Word-color	8
Gschwind et al. (2015)	153	H	74.7	IStoppFalls system and educational material	16 weeks, 180 min per week	Educational material	Stroop Trail Making Digit Span Flanker	9
Phirom, Kamnardsiri & Sungkarat (2020)	39	H	69.8	Interactive game-based training	12 weeks, 3 × 60 min per week	Educational material	Trail Making	8
Guimarães, Barbosa & Meneghini (2018)	27	H	60.4	Kinect Sports Ultimate Collection	12 weeks, 3 × 60 min per week	Aerobic exercise	Groton Maze Learning	8
Schättin et al. (2016)	27	H	79.2	Cognitive-motor training	8 weeks, 3 × 30 min per week	Conventional balance training	Go/No go Set-shifting	8
Schoene et al. (2015)	81	H	81.5	Interactive Step Training	16 weeks, 3 × 20 min per week	Educational material and continue usual activities	Stroop Trail Making Digit Span Flanker	8
Ordnung et al. (2017)	29	H	69.2	Summer Stars 2012	6 weeks, 2 × 60 min per week	No training	N-back Response inhibition	7
Liao et al. (2019)	34	MCI	74.3	VR-based physical and cognitive training	12 weeks, 3 × 60 min per week	Traditional physical and cognitive training	Stroop Trail making	8
Liao et al. (2020)	34	MCI	74.3	VR-based physical and cognitive training	12 weeks, 3 × 60 min per week	Traditional physical and cognitive training	Executive Interview 25	8
Monteiro-Junior et al. (2017a)	19	H	86	exercises with VR	a single session, 30–45 min	Exercises without VR	Digit Span	7
Monteiro-Junior et al. (2017b)	18	H	85.5	VR-based physical exercise with exergame	6–8 weeks 2 × (30–40) sessions per week	Exercises without VR	Trail Making Floor Maze	8
Adcock et al. (2020)	31	H	73.9	Active @ Home	16 weeks, 3 × (30–40) min per week	Normal daily living	Stroop Trail Making Digit Span	8
Schoene et al. (2013)	30	H/MCI	78	Computerized step pad system	8 weeks, (2–3) × (30–40) min per week	Continue usual activities	Trail Making	8

**Note:**

MCI, mild cognitive impairment; H, healthy; VR, virtual reality.

### Effects of exergaming interventions on EF

**Figure 2:**
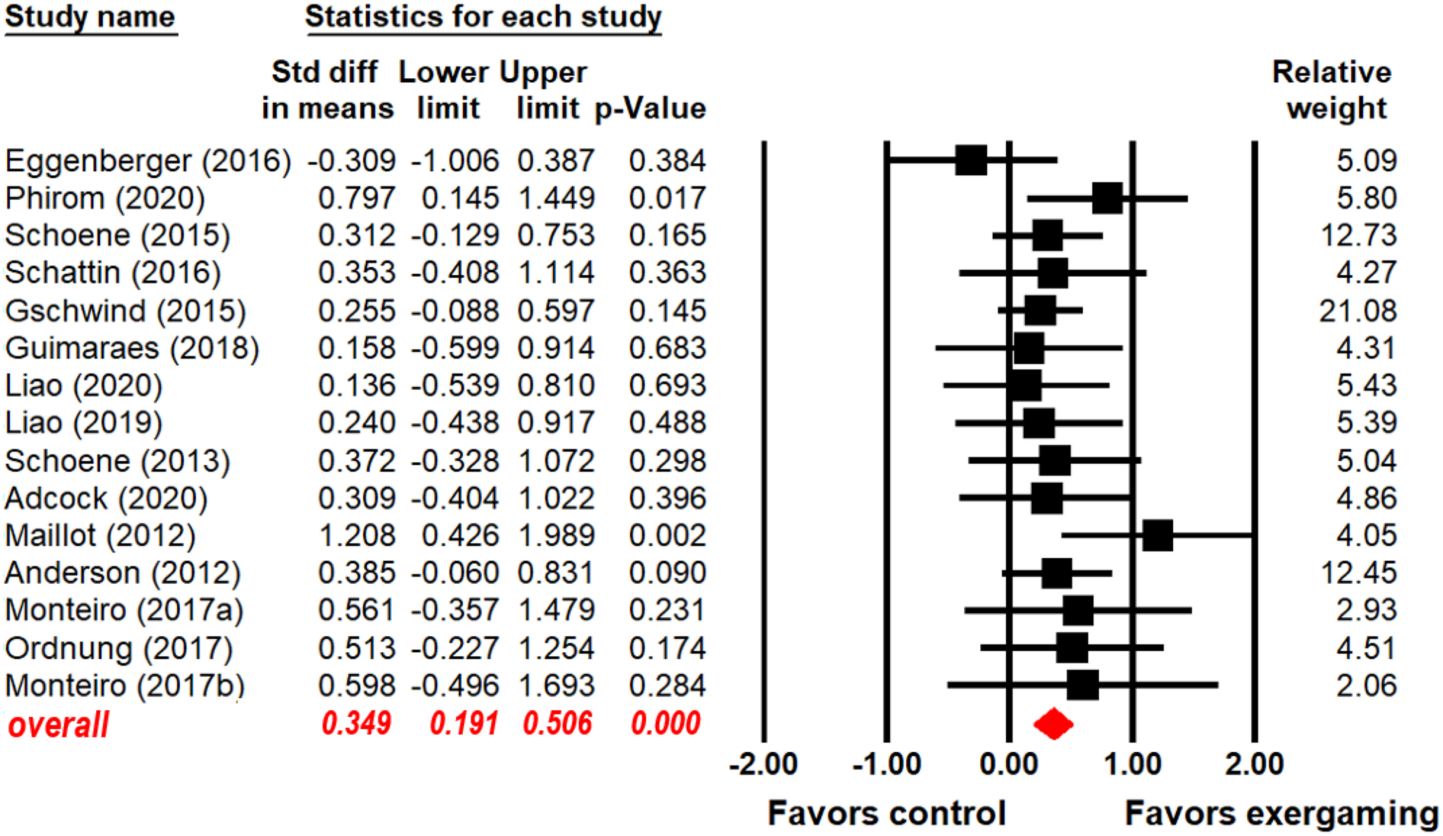
Forest plot for the effect sizes of exergaming on overall EF.

As shown in Fig. 2 (key statistical values in red), participants who were subjected to an exergaming intervention had better overall EF than control subjects (SMD = 0.349, 95% CI [0.191–0.506], *p* < 0.001). A heterogeneity test revealed no significant heterogeneity between exergaming and control group (Q_(14)_ = 11.569, I^2^ = 0.000, *p* = 0.641).

**Figure 3:**
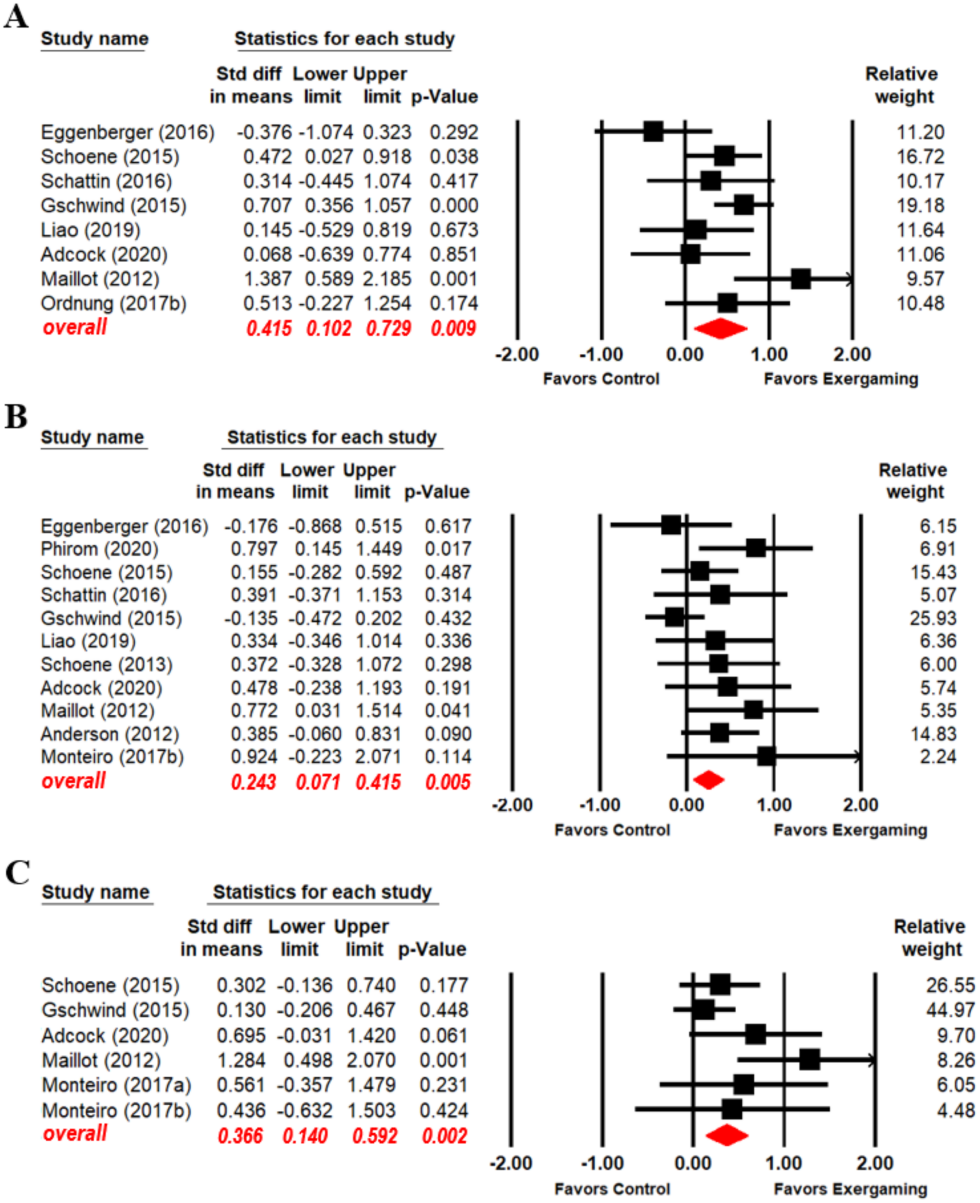
Forest plot for the effect sizes of exergaming on inhibition (A), switching (B) and updating (C).

Because exergaming effect sizes on EF domains were unclear, we explored exergaming effects on EF domains separately. Eight (Maillot, Perrot & Hartley, 2012; Schoene et al., 2013; Gschwind et al., 2015; Eggenberger et al., 2016; Schättin et al., 2016; Ordnung et al., 2017; Liao et al., 2019; Adcock et al., 2020), eleven (Maillot, Perrot & Hartley, 2012; Anderson-Hanley et al., 2012; Schoene et al., 2013, 2015; Gschwind et al., 2015; Eggenberger et al., 2016; Schättin et al., 2016; Monteiro-Junior et al., 2017a; Liao et al., 2019; Adcock et al., 2020; Phirom, Kamnardsiri & Sungkarat, 2020), and six (Maillot, Perrot & Hartley, 2012; Gschwind et al., 2015; Schoene et al., 2015; Monteiro-Junior et al., 2017a, 2017b; Adcock et al., 2020) articles reported exergaming intervention effect data for the EF domains of inhibition, switching, and updating, respectively. Subjects who participated in exergaming performed better than control subjects in inhibition (SMD = 0.415, 95% CI [0.102–0.729], *p* = 0.009), switching (SMD = 0.243, 95% CI [0.071–0.415], *p* = 0.005), and updating (SMD = 0.366, 95% CI [0.140–0.592], *p* = 0.002) (Fig. 3; key statistical values in red). Significant heterogeneity between the exergaming and control groups was found in the inhibition analysis (Q_(7)_ =14.771, I^2^ = 52.611, *p* = 0.039). No significant heterogeneity between the two groups in the switching analysis (Q_(10)_ = 13.639, I^2^ = 26.683, *p* = 0.19) and updating analysis (Q_(5)_ = 8.179, I^2^ = 38.866, *p* = 0.147).

### Moderator analysis

The results of the moderator analysis—including SMD values, 95% CIs, and homogeneity test statistical values—are summarized in Table 2. The following variables were analyzed: mean participant age (high or low); cognitive status (healthy or MCI); sample size (big or small); sample size (smaller or larger); control group type (active or passive); intervention duration (in weeks; long, medium, or short); intervention session frequency (high or low); exergaming type (off-the-shelf and customized) and session duration (long, medium, or short). Among these variables, intervention frequency emerged as a significant moderator of the effects of exergaming on overall EF in older adults.

**Table 2:** Moderator analysis for the exergaming group *vs* the control group.

Moderator	Level	No. of studies	SMD	95% CI	I^2^	Homogeneity test		
						*Q*	df	*p*
Mean age	High (≥75 years old)	6	0.381**	[0.130–0.631]	0	0.104	1	0.747
	Low (<75 years old)	9	0.328**	[0.126–0.530]	27.703			
Sample size	Big (>35 people)	4	0.360**	[0143–0.578]	0.000	0.024	1	0.877
	Small (≤35 people)	11	0.336**	[0.109–0.563]	0.000			
Cognitive status	Healthy	11	0.365**	[0.179–0.550]	9.308	0.460	1	0.497
	MCI	2	0.187	[−0.290 to 0.665]	0.000			
Intervention duration	Long (>12 weeks)	4	0.368**	[0.128–0.609]	39.355	0.153	2	0.926
	Medium (>8 to ≤12 weeks)	5	0.363**	[0.091–0.635]	0.000			
	Short (≤8 weeks)	6	0.293	[−0.029 to 0.614]	0.000			
Frequency	High (≥3 sessions/week)	9	0.291**	[0.088–0.493]	0.000	3.537	1	0.060
	Low (<3 sessions/week)	4	0.744**	[0.317–1.171]	0.000			
Session duration	Long (≥60 min)	5	0.550**	[0.237–0.863]	27.803	2.577	2	0.276
	Medium (>30 to <60 min)	5	0.404**	[0.104–0.705]	0.000			
	Short (≤30 min)	3	0.177	[−0.158 to 0.511]	18.023			
Control group	Active	11	0.298**	[0.124-0.472]	0.000	1.775	1	0.183
	Passive	4	0.573**	[0.207–0.939]	11.893			
Exergaming type	Off-the-shelf	9	0.322**	[0.141–0.502]	0.000	0.356	1	0.551
	Customized	6	0.433**	[0.114–0.753]	9.229			

**Notes:**

** *p* < 0.01.

MCI, mild cognitive impairment.

Exergaming effects on overall EF differed across age, cognitive status, sample size, exergaming types and control groups. In terms of mean age, significant effects were found both for high age participants (SMD = 0.381, 95% CI [0.130–0.631], *p* = 0.003) and low age (SMD = 0.328, 95% CI [0.126–0.530], *p* = 0.001) participants. Regarding cognitive status, exergaming had significant effects with healthy participants (SMD = 0.365, 95% CI [0.179–0.550], *p* < 0.001), but not with MCI participants (SMD = 0.187, 95% CI [−0.290 to 0.665], *p* = 0.442). Significant intervention effects were observed both with larger (SMD = 0.360, 95% CI [0.143–0.578], *p* = 0.001) and smaller (SMD = 0.336, 95% CI [0.109–0.563], *p* = 0.004) sample sizes. In terms of control group, significant effects of the intervention were observed on overall EF both in active control groups (SMD = 0.298, 95% CI [0.124–0.472], *p* = 0.001) and passive control groups (SMD = 0.573, 95% CI [0.207–0.939], *p* = 0.002). Regarding exergaming type, significant effects were found both for off-the-shelf exergaming (SMD = 0.322, 95% CI [0.141–0.502], *p* < 0.001) and customized exergaming (SMD = 0.433, 95% CI [0.114–0.753], *p* = 0.008).

Exergaming effects on overall EF differed in relation to intervention duration, session frequency, and session duration. Significant heterogeneity was observed between the two frequency subgroups (Q_(1)_ = 3.537, *p* = 0.06) (Table 2). Specifically, the exergaming intervention was found to have significant effects when administered both for high-frequency (SMD = 0.291, 95% CI [0.088–0.493], *p* = 0.005) and low-frequency (SMD = 0.744, 95% CI [0.317–1.171], *p* = 0.001). Regarding intervention duration, exergaming had significant effects on EF when the intervention duration had a long (SMD = 0.368, 95% CI [0.128–0.609], *p* = 0.003) or medium (SMD = 0.363, 95% CI [0.091–0.635], *p* = 0.009) duration, though a larger effect size was generated with long duration interventions than with medium duration interventions. No significant effect of exergaming protocol with short intervention duration was observed (SMD = 0.293, 95% CI [−0.029 to 0.614], *p* = 0.074). Regarding session duration, exergaming had significant effects on EF when the session had a long (SMD = 0.550, 95% CI [0.237–0.863], *p* = 0.001) or medium (SMD = 0.404, 95% CI [0.104–0.705], *p* = 0.008) duration, though a larger effect size was generated with medium duration interventions than with long duration interventions. No significant effect of exergaming protocol with short-duration sessions was observed (SMD = 0.177, 95% CI [−0.158 to 0.511], *p* = 0.301).

## Discussion

The present quantitative meta-analysis of 12 studies showed that exergaming has positive effects on EF in older adults. Benefits of exergaming were confirmed for overall EF as well as for the inhibition, switching, and updating EF domains. Furthermore, the effects of exergaming on overall EF were found to be moderated by intervention frequency.

The present findings complement the findings of a prior meta-analysis demonstrating positive effects of exergaming on balance (Fang et al., 2020) and postural control (Tahmosybayat et al., 2017) in elderly participants. These findings support the view that combined physical and cognitive interventions can improve EF in older adults effectively (Guo et al., 2020). Notably, in the present study, we examined overall EF as well as specific EF domains, namely inhibition, switching, and updating. The present data showed a general enhancing effect of exergaming on overall EF in elderly participants. Exergaming can be considered as one kind of activities that train and challenge diverse motor and EF skills, bring joy, pride, and self-confidence (Diamond, 2015). In this view, it is easy to understand the general enhancing effect of exergaming on overall EF, older adults should be encouraged to go beyond simply moving to moving with thought in older to improve EF. Significant improvements were found in each of the three aforementioned aspects of EF, with the largest effect size on the inhibition. Inhibition is recognized as a sensitive function affected by exergaming (Dhir et al., 2021), which requires participants to restraint or suppression the inappropriate process or response continually. So, the exergaming obviously is a good exercise for inhibition. The effect of exergaming on the switching and updating were also significant, it probably due to the variability of the game environment. Participants were required to switch between different tasks and rules and update their existing memory resources in order to quickly adapt to the changing environment and achieve better exergaming performance (Gschwind et al., 2015; Adcock et al., 2020).

Interestingly, the moderator analysis indicated that low-frequency exergaming programs (<3 sessions/per week) resulted in significant overall EF improvements, whereas high-frequency interventions (≥3 sessions/per week) resulted in smaller effect. It is consistent with results in previous studies reporting significant effects of low-frequency interventions on cognitive function in older people (Lampit, Hallock & Valenzuela, 2014; Wang, Zhou & Shah, 2014; Zhu et al., 2016). This is probably due to the fact that tightly intervention might cause cognitive fatigue, which may reduce the engagement levels of the intervention in older adults. More distributed training could produce larger gains than high-frequency interventions. Appropriate frequency of intervention may be crucial to EF improvement in older people.

Additionally, we found stronger intervention effects when there were active control groups than when there were passive control groups. Active control groups are presumed to have more prescient control of experimental variables than passive control groups, and thus demonstrations of effects compared to an active control group may make a stronger case for efficacy than can be made based on comparisons to a passive control group.

In the analysis of intervention effects on the EF domain of inhibition, an apparent heterogeneity was observed between the exergaming group and the control group. Although one of the eight studies reporting inhibition data reported an effect size that exceeded 3 SDs, suggesting it may be an outlier, the funnel plot did not indicate significant asymmetry (Egger’s regression intercept = −1.43, *p* > 0.05). Therefore, that study was retained in the analyses. Notwithstanding, when we did conduct a parallel analysis with the questioned study excluded, the significant effects of exergaming on EF remained, suggesting that the intervention has a stable influence of EF in elderly participants.

EF contributes to the overall quality of life of older adults. Improved EF has been associated with reduced risk of falls (Lange et al., 2010; Adcock et al., 2019), which can lead to serious physical injury and psychological damage (Jung, 2008; Peel, 2011). Regarding methods of EF enhancement, exergaming is more relevant to daily life than single-domain interventions because most daily life activities require the simultaneous performance of cognitive and physical functions. Thus, exergaming may be a very promising tool for supporting and enhancing the health status of older people.

This study had three notable limitations. First, the statistical power of the meta-analysis was limited by there being relatively few studies that have examined the effects of exergaming on EF in older adults in the literature. Second, the reliability of the results was limited by the sample sizes of the studies included. The need for larger sample sizes was indeed one of the reasons why we conducted this meta-analysis. Third, the exergaming employed differed among the analyzed studies. A standard classification scheme should be established for exergaming types in future studies.

## Conclusions

The findings of this meta-analysis indicate that exergaming has significant EF benefits in older adults. Furthermore, the effects of exergaming on overall EF were found to be moderated by the frequency of the intervention.

## Supplemental Information

10.7717/peerj.13194/supp-1 Supplemental Information 1 Raw data. Means and standard deviations EF outcome in each group at baseline and post-intervention.

10.7717/peerj.13194/supp-2 Supplemental Information 2 Results of literature quality assessment of included studies.

10.7717/peerj.13194/supp-3 Supplemental Information 3 PRISMA checklist.

10.7717/peerj.13194/supp-4 Supplemental Information 4 Rationale and contribution of systematic review.

## Additional Information and Declarations

### Data Availability

The following information was supplied regarding data availability: The raw measurements are available in the Supplemental Files.
