# Supplementary material for: Effects of exergaming on executive function of older adults: a systematic review and meta-analysis
Source: PeerJ. 2022 Apr 11;10:e13194. doi: 10.7717/peerj.13194 (PMC9009327; doi:10.7717/peerj.13194)
Supplement: Supplemental Information 4 [file peerj-10-13194-s004.docx]

**E****ffects of exergaming on executive function of older adults: A systematic review and meta-analysis**

**1. Rationale for conducting the systematic review.**

Aging-related cognitive decline has been shown to be strongly associated with executive function (EF) worsening. Several interventions were used to improve EF in older adults. Exergaming is an emerging simultaneous intervention that combines physical exercise with video games. Among the prior studies that have been reported on the effects of exergaming on EF in older adults, there are inconsistencies among them in terms of intervention design as well as inconsistencies in the cognitive status of the subjects. Additionally, there have been meta-analyses that have examined the effects of exergaming on balance and postural control in older adults, but there have not been any that have examined the effects of exergaming on EF in older adults. Therefore, the current study was to perform a meta-analysis evaluating the effects of exergaming on EF performance in older adults and to conduct a moderator analysis of participant and intervention characteristic variables.

**2. The contribution that the systematic review makes to knowledge in light of previously published related reports.**

Practically, a promising tool was provided in this study to improve executive function for older adults. In this study, benefits of exergaming were confirmed for overall EF as well as for the inhibition, switching, and updating EF domains. Also, the effects of exergaming on overall EF were found to be moderated by intervention duration. Further researches on the effects of exergaming on executive function should pay more attention to the selection of the appropriate intervention duration. Additionally, a standard classification scheme was suggested to be established for exergaming types in future studies due to the unclear division of exergaming types in the included studies.
